# Treponema pallidum Subspecies *Pallidum* Intrapatient Homogeneity at Various Body Locations in Men with Infectious Syphilis

**DOI:** 10.1128/spectrum.02482-21

**Published:** 2022-06-23

**Authors:** H. C. A. Zondag, S. A. Nieuwenburg, M. Himschoot, A. P. van Dam, M. F. Schim van der Loeff, H. J. C. de Vries, S. M. Bruisten

**Affiliations:** a Department of Infectious Diseases, Public Health Service Amsterdam, Amsterdam, the Netherlands; b Amsterdam Institute for Infection and Immunity, Amsterdam UMC, University of Amsterdam, Amsterdam, the Netherlands; c Department of Internal Medicine, Division of Infectious Diseases, Amsterdam UMC, University of Amsterdam, Amsterdam, the Netherlands; d Department of Medical Microbiology, Amsterdam UMC, University of Amsterdam, Amsterdam, the Netherlands; e Department of Dermatology, Amsterdam UMC, University of Amsterdam, Amsterdam, the Netherlands; University of Utah and ARUP Laboratories

**Keywords:** Treponema pallidum, molecular epidemiology, molecular subtyping, infectious disease, sexually transmitted disease, molecular variation, syphilis

## Abstract

Syphilis, caused by Treponema pallidum subspecies *pallidum* (TP), is a complex multistage infectious disease. Systematic dissemination occurs within a few hours of transmission. We determined the molecular variation of TP at various body locations and peripheral blood within patients in different stages of syphilis to assess the distribution of TP strains at these locations. We included 162 men who have sex with men (MSM) with syphilis visiting the Sexual Health Center in Amsterdam between 2018 to 2019, who had TP DNA detected in at least one sample type (anal swab, urine sample, peripheral blood, pharyngeal swab, and/or ulcer swab). TP DNA was detected in 287 samples using a qPCR targeting the *polA* gene. With multilocus sequence typing (TP-MLST) based on partial sequence analysis of three genetic regions (*tp0136, tp0548, tp0705*), we characterized all TP DNA positive samples. Samples could be typed (119/287) from at least one anatomical location or peripheral blood from 93/162 (57%) patients in the following stages: 48 (52%) primary, 35 (38%) secondary, and 10 (11%) early latent stage syphilis. The TP-MLST type was identical within each of the 12 patients with typed samples at ≥2 different body locations. The most prevalent TP strains were 1.3.1 (39/93, 42%) and 1.1.1 (17/93, 18%) belonging to the SS14 lineage; 80% (74/93) of the patients carried a SS14 lineage TP strain and 20% (19/93) Nichols lineage. The distribution of TP-MLST types did not differ between patients by syphilis stage. We found intrapatient TP strain homogeneity and no TP strain variation between anatomical location or syphilis stages. More early latent samples should be typed and added in future studies to investigate this in more detail.

**IMPORTANCE** Syphilis, caused by Treponema pallidum subspecies *pallidum*, is a complex multistage infectious disease. Systematic dissemination is known to occur within a few hours of transmission. Despite the effective antibiotic penicillin, syphilis remains prevalent worldwide. Men who have sex with men are disproportionally affected in high income countries like the Netherlands where 96% of the syphilis cases in 2020 were among this population. The inability to *in vitro* culture T. pallidum directly from patient samples limits whole-genome sequencing efforts. Fortunately, in 2018 a multilocus sequence typing technique was developed for T. pallidum allowing the monitoring of circulating strains. The significance of our research is in the investigation of T. pallidum molecular variation at various body locations and blood within patients in different stages of syphilis in order to assess the distribution of strains at these locations.

## INTRODUCTION

Syphilis, caused by the bacterium Treponema pallidum subspecies *pallidum* (TP), is a complex multistage sexually transmitted disease. Although highly effective treatment is available, the worldwide prevalence of syphilis remains an important public health issue. Like in many high-income countries, in the Netherlands men who have sex with men (MSM) are most affected. In 2020, 96% of the syphilis diagnoses are among MSM (1).

Systemic dissemination occurs within a few hours after acquisition (2). Sexual transmission typically occurs during the primary, secondary, and early latent stages within the first year of acquisition. During early syphilis stages, syphilis lesions are considered infectious. Yet, recent studies have detected TP DNA at various seemingly nonaffected tissues, and in peripheral blood (3, 4). Body locations and fluids that are considered pivotal in sexual transmission of TP, such as genital, anal, and pharyngeal cavities were tested for the presence of TP DNA by PCR. It is unclear whether TP DNA presence at multiple locations is the result of a primary infection at one body site followed by systemic dissemination, or a consequence of multiple sexual exposures at different anatomical locations. Molecular typing of TP may be helpful to elucidate this (5, 6).

Bacterial typing is usually most successful on cultured strains, from which high amounts of specific bacterial DNA can be isolated. Despite recent technological advances, direct *in vitro* culture of TP from patient samples remains unsuccessful (7). Culture independent molecular characterization of TP isolates using the multilocus sequence typing (MLST) method allows strain identification of TP for comparisons between patients (6), but this technique may also be useful to discriminate TP types within patients.

The aim of this study was to investigate possible intrapatient TP strain variation and to assess possible associations with specific body locations. In addition we aimed to investigate the molecular variation of TP in different stages of syphilis.

## RESULTS

### Patient characteristics.

We included 162 patients, of whom the majority (99%) had exclusively sex with men (Table 1) and almost one third (31%) were living with HIV. In 24 of the 162 patients (15%), who tested positive for TP DNA in at least one body location or peripheral blood, the rapid plasma reagin (RPR) was negative. Of these, 22 patients were diagnosed with primary syphilis and two with early latent syphilis determined by a seroconversion of the chemoluminence immunoassay (CLIA).

**TABLE 1:** Sociodemographic, behavioral, and clinical characteristics of men who have sex with men (*N* = 162) included in this study in Amsterdam, the Netherlands, 2018 to 2019^*a*^

Age	
<35 yrs	58 (36%)
35–44 yrs	48 (30%)
≥45 yrs	56 (35%)
Education	
Primary/secondary	35 (22%)
College/university	106 (65%)
Unknown	21 (13%)
Gender of sex partners	
Men	160 (99%)
Men and women	2 (1%)
No. of sex partners (6 mo)^*b*^	
Median (IQR)	6 (4 to 15)
<5	46 (28%)
5 to 9	45 (28%)
10 to 14	24 (15%)
≥15	44 (27%)
Unknown	3 (2%)
HIV status	
Negative	112 (69%)
Positive	50 (31%)
cART use if HIV positive	
No	3 (6%)
Yes	47 (94%)
Most recent CD4 count (cells/μL)	
<350	0 (0%)
350 to 499	2 (4%)
≥500	33 (66%)
Unknown	15 (30%)
RPR titre	
Median [IQR]	16 (2 to 32)
Negative^*c*^	24 (15%)
1:1 to 1:4	30 (19%)
1:8 to 1:16	56 (34%)
1:32 to 1:128	52 (32%)

a IQR, interquartile range; HIV, human immunodeficiency virus; cART, combination antiretroviral therapy; RPR, Rapid Plasma Reagin test.

b Number of sex partners in the 6 months before the consultation.

c Of these 24 patients, 22 had primary syphilis with an ulcer and two without symptoms, but with a seroconversion of the chemoluminence immunoassay.

### Sample characteristics.

From the 162 included patients we collected 287 TP DNA positive samples (Table S1). Among these samples were 73 ulcers of which 16 were ulcers found at the anal site, 55 at the urogenital site, and two at the pharyngeal site. TP DNA was detected most frequently at the anal site, peripheral blood, and the pharyngeal site among the patients with secondary syphilis compared with the other syphilis stages. Samples from the urogenital site were most frequently positive among patients with primary syphilis. There was only one peripheral blood sample that could be fully typed. Over two thirds of the patients with secondary or early latent syphilis had a positive TP DNA sample at the pharynx (Table 2).

**TABLE 2:** An overview of TP DNA detected and typed at the different anatomical locations and peripheral blood from the 162 included patients between 2018 and 2019 at the SHC in Amsterdam, the Netherlands by syphilis stage

Anatomical location and blood	Primary syphilis		Secondary syphilis		Early latent syphilis		Total	
	*N* = 69 patients		*N* = 64 patients		*N* = 29 patients		*N* = 162 patients	
	TP DNA detected	Typed	TP DNA detected	Typed	TP DNA detected	Typed	TP DNA detected	Typed
	*n*	*n*	*n*	*n*	*n*	*n*	*n*	*n*
Anus	16 (23%)	9 (13%)	37 (58%)	7 (11%)	11 (38%)	1 (3%)	64 (40%)	17 (11%)
Urogenital	55 (80%)	38 (55%)	28 (44%)	12 (19%)	6 (21%)	2 (7%)	89 (55%)	52 (32%)
Peripheral blood	2 (3%)	0 (0%)	15 (23%)	1 (2%)	5 (17%)	0 (0%)	22 (14%)	1 (1%)
Pharynx	7 (10%)	4 (6%)	48 (75%)	24 (38%)	21 (72%)	9 (31%)	76 (47%)	37 (23%)

**FIG 1:**
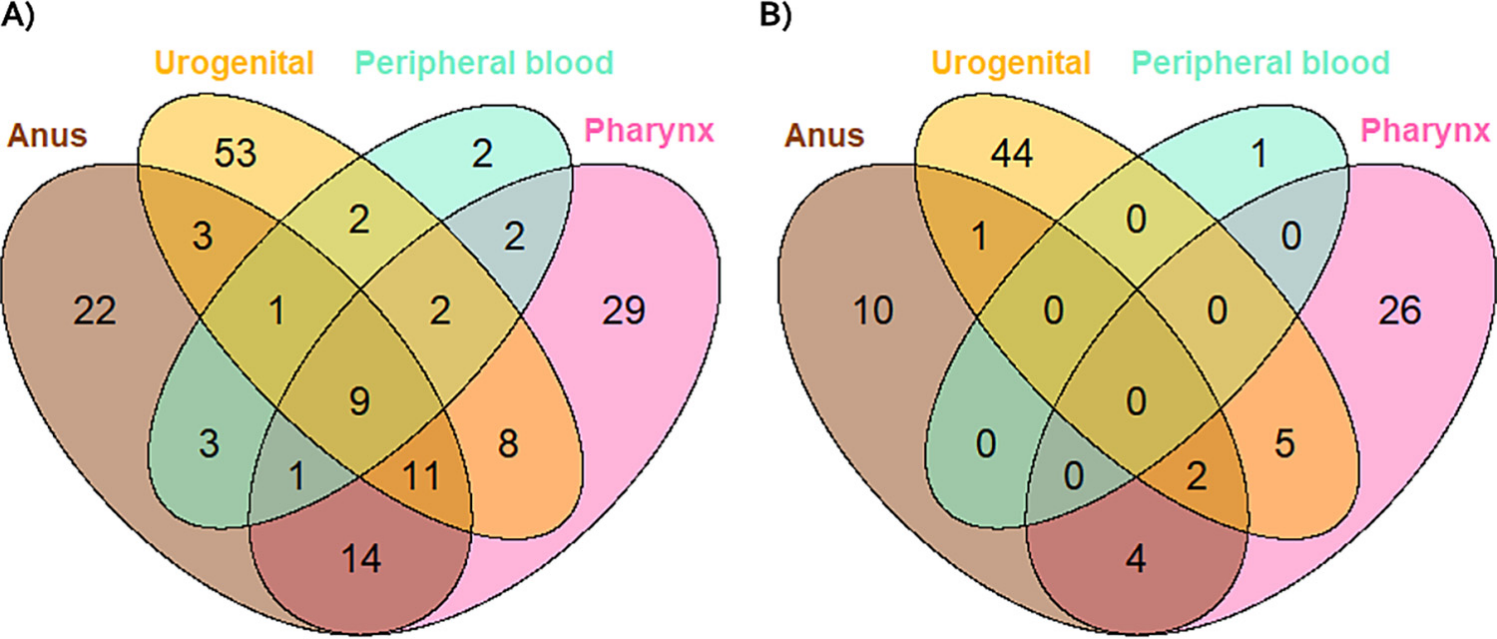
Venn Diagrams showing the number of patients with (A) TP DNA detected by anatomical location and peripheral blood (B) where a TP type was obtained in 93 patients with at least one typed sample.

In 9/162 (6%) patients, TP DNA was detected in samples from all sites, including peripheral blood (Fig. 1A). Also, a third (53/162) of the patients were positive only at the urogenital site (Fig. 1A).

### TP typing at the distinct anatomical locations and peripheral blood.

TP-MLST types were successfully obtained for 119/287 (42%) samples (Table S1), which were grouped to 107/251 (43%) anatomical locations and peripheral blood samples (Table 2) and derived from 93 patients (Fig. 1B). Among these 93 patients, 48 (52%) patients had primary syphilis, 35 (38%) secondary syphilis, and 10 (11%) early latent syphilis. In 12 patients, two or more locations were successfully typed (Fig. 1B). From all but one of these patients, a TP-MLST type was obtained from the pharyngeal site in combinations with samples from other sites (11/12, 92%). Samples from the urogenital site were most often successfully typed, followed by samples from the pharynx.

The cycle threshold (Ct) value of the initial *polA* TP DNA PCR was significantly lower in samples that were successfully typed (Fig. S1A). However, the distribution of Ct values overlapped substantially between the typeable and nontypeable samples. In addition, the Ct value was found to vary greatly by sample type (Fig. S1B).

### TP strain distribution per syphilis stage.

The 107 distinct anatomical locations and peripheral blood samples containing typed samples from 93 patients resulted in 14 different allelic profiles (Table 3). Most prevalent allelic profiles were 1.3.1 and 1.1.1 occurring in 39 (42%) and 17 (18%) patients, respectively. Eight of the 14 allelic profiles (57%) were each found in only one patient. Nine distinct allelic profiles were found among the 48 patients with primary syphilis, 10 among patients with secondary syphilis, and five among patients with early latent syphilis. The distribution of TP-MLST types did not differ between patients by syphilis stage (*P* = 0.31, Fisher’s exact test).

**TABLE 3:** TP strain distribution per syphilis stage among the 93 typed patients with at least one typed sample out of the 162 included patients between 2018 and 2019 at the SHC in Amsterdam, the Netherlands

	Allelic profiles by syphilis stage				
Allelic profiles	Primary	Secondary	Early latent	Total (%)	Lineage
1.3.1	25	11	3	39 (42)	SS14
1.1.1	9	5	3	17 (18)	SS14
9.7.3	3	7	1	11 (12)	Nichols
1.1.8	3	3	2	8 (9)	SS14
3.2.3	4	3	0	7 (8)	Nichols
1.1.9	1	2	0	3 (3)	SS14
1.64^*a*^.1	1	0	0	1 (1)	SS14
1.52.1	1	0	0	1 (1)	SS14
1.17.9	1	0	0	1 (1)	SS14
1.43.1	0	0	1	1 (1)	SS14
1.66^*a*^.1	0	1	0	1 (1)	SS14
1.65^*a*^.1	0	1	0	1 (1)	SS14
30^*a*^.3.1	0	1	0	1 (1)	SS14
29^*a*^.7.3	0	1	0	1 (1)	Nichols
Total	48	35	10	93	

a New allelic variants found in this study.

**FIG 2:**
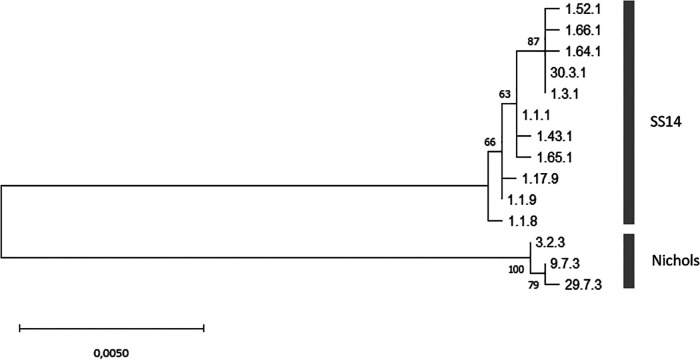
Phylogenetic tree of concatenated sequence (MUSCLE alignment of 2,584 bp) of all allelic profiles found in this study using the maximum likelihood methods and Tamura-Nei model with 1,000 bootstraps.

The overall typing success per genetic region was 148/287 (52%) for *tp0136*, 144/287 (50%) for *tp0548*, and 160/287 (56%) for *tp0705*. Besides the 119 fully typed samples, 32/287 (11%) samples were typed for two genetic regions and 59/287 (21%) for only one genetic region (Table S2). 77/287 (27%) samples were not successfully typed for any of the regions. We found six new allelic variants of which two for genetic region *tp0136* and 5 for genetic region *tp0548*. This resulted in five new allelic profiles as one new allelic variant was part of a partially typed sample. One of the new allelic variants for *tp0136*, in the profile 29.7.3, belongs to the Nichols lineage (Fig. 2). All other new allelic profiles belong to the SS14 lineage. The new allelic variants and profiles were uploaded in the pubMLST database (8) and were subsequently numbered (*tp0136*: 29, 30 and *tp0548*: 63, 64, 65, 66).

In Fig. 2, the diversity of concatenated sequences is shown from the allelic profiles found in this study (an alignment of 2,584 bp) and the distinction between the SS14 and Nichols lineages. The three types belonging to the Nichols lineage were found in 19/93 (20%) patients. Strains from neither the SS14 nor the Nichols lineage were found to be overrepresented in patients with a specific syphilis stage (Table S3A). In addition, no differences were found in the number of Nichols or SS14 lineages per age group, HIV status or the number of sites in which TP DNA was detected within the 93 patients (Tables S3B, 3C, 3D) Single-nucleotide polymorphism (SNP) level variation was found within these lineages.

In patients with successfully typed samples from multiple different body locations, allelic profiles were identical within each patient (Table 4). None of these patients had a peripheral blood sample that was successfully typed. All patients had a positive RPR titer and, similar to the overall included population, four of these 12 patients (33%) were living with HIV. 5/12 (42%) patients reported having had fewer than five sex partners in the past 6 months (Table 4).

**TABLE 4:** Overview of patients (*n* = 12) with two or more obtained TP-MLST types at different anatomical locations among the 162 included patients between 2018 and 2019 at the SHC in Amsterdam, the Netherlands

		Demographic and clinical characteristics			Obtained TPA strain per body site (x)^*b*^				
Patient nr.	Syphilis stage	No. of sex partners (6 mo)^*c*^	HIV status	RPR titer	Anus	Urogenital	Pharynx	Allelic profile	Lineage
1	Primary	1	Negative	32	x		x	1.1.1	SS14
2	Primary	12	Negative	8		x	x	1.3.1	SS14
3	Primary	10	Positive	16	x		x	9.7.3	Nichols
4	Secondary	2	Positive	8	x		x	1.1.1	SS14
5	Secondary	2	Positive	32	x	x		1.1.9	SS14
6	Secondary	10	Negative	16		x	x	1.3.1	SS14
7	Secondary	7	Positive	32	x		x	1.66^*a*^0.1	SS14
8	Secondary	5	Negative	16	x	x	x	9.7.3	Nichols
9	Secondary	5	Negative	16	x	x	x	9.7.3	Nichols
10	Secondary	5	Negative	32		x	x	9.7.3	Nichols
11	Early latent	3	Negative	32		x	x	1.1.1	SS14
12	Early latent	2	Negative	8		x	x	1.3.1	SS14

a New allelic variant.

b There were no peripheral blood samples typed in these 12 patients.

c In the 6 months before consultation.

## DISCUSSION

This is the first study that applied MLST to TP DNA positive samples collected at different anatomical locations and from peripheral blood from patients in different syphilis stages. We found no TP strain variance within a patient. This study focused on the early syphilis stages, primary, secondary, and early latent, as we did not detect TP DNA in samples from participants with late stage syphilis or treated syphilis. TP strains were not associated with a specific syphilis stage.

The intrapatient TP strain homogeneity suggests that the TP DNA detected at the different body locations is due to haematogenous dissemination rather than sexual transmission from multiple partners. Direct transmission of multiple different body locations during the same sex act could also explain the intrapatient homogeneity. However, previous studies that included molecular typing techniques on multiple samples of TP isolates within a patient showed identical types in blood samples and swab samples supporting the suggestion of haematogenous dissemination (9, 10). In addition, due to the clonal nature of TP the possibility of infection from multiple partners with the same allelic profile, such as the highly prevalent strain, 1.3.1, also exists. More in-depth typing methods such as whole-genome sequencing (WGS) might reveal more polymorphism for intrapatient TP strains.

The significant difference between cycle threshold values of successfully typed samples versus not (fully) typed samples was expected as Ct values are associated with the bacterial load in the sample, and thus influence amplification success of each genetic locus. We successfully typed 119/287 (43%) samples in this study. This is a relatively low percentage compared with other studies on TP using this MLST (6, 11–15), but in these studies mainly ulcer swabs were included, whereas we also included many samples from unaffected tissues and peripheral blood samples, many with high Ct values. The number of multiple typed anatomical locations within patients was therefore limited. Due to the higher TP DNA load in swab samples from ulcers, a higher proportion of patients with primary syphilis had at least one sample successfully typed.

The TP strain diversity was similar to that in previous TP-MLST studies among MSM in Amsterdam, the Netherlands (11). The most prevalent allelic profiles were 1.3.1 (42%) and 1.1.1 (18%), which have been found in all TP-MLST studies (8). With the current knowledge, the unique TP strains within countries (6, 11–13, 15) seem to suggest local transmission besides international mixing as was also found in this study with 8/14 (57%) allelic profiles occurring only once. The 20% prevalence of TP strains belonging to the Nichols lineage in this study is similar to previous studies in Amsterdam (11), Japan (16), and Argentina (17), and is comparable with a recent large-scale genomic study of samples from 23 countries (18). However, a large older study on 970 publicly available sequences described that 93.5% belonged to the SS14 lineage (19) and some countries showed <10% circulating Nichols strains, such as in the Czech Republic (12), France (13), and Switzerland (6). No associations were found between TP strains and syphilis stage (Table S3).

Our study has a number of limitations. The molecular method with the highest resolution for the analysis of molecular variation within a patient or between syphilis stages would be WGS. However, current TP WGS efforts using target enrichment of pathogen reads show that TP bacterial loads are crucial and samples with lower bacterial loads, like the majority from this study, would be unsuccessful using WGS (19–21). The Enhanced Centers for Disease Control and Prevention Typing method (ECDCT) is a different typing method for TP, combining the analysis of tandem repeats, restriction fragment length patterns, and sequence analysis (5). This ECDCT method was previously found to contain genetically unstable loci (9, 22). However, recently this technique was found to remain stable and distinguish typing within different TP-MLST types and vice versa (10). After WGS, the TP MLST provides high resolution with a discriminatory power of 30.8% in comparison with WGS (6).

Also, we had a low number of typed samples from the early latent syphilis stage, because of the lower TP DNA positivity in patients with this stage. Nonetheless, we were able to type samples in 10 patients with early latent syphilis, of whom two had a type that was obtained from both the urogenital site and the pharyngeal site.

The monitoring of molecular changes keeps us updated on the circulating TP strains among populations at higher risk of a syphilis infection. With help of the TP pubMLST database (8), the knowledge on genetic information of TP strains is expanded internationally. In this study, we found intrapatient TP strain homogeneity and no TP strain variation between anatomical locations or syphilis stages, although more early latent samples and more within patient samples should be typed in future studies to investigate this in more detail.

## MATERIALS AND METHODS

### Ethics statement.

This study was approved by the Medical Ethics Committee of the Amsterdam University Medical Centers (reference: NL66419.018.18, 2018_236#B2018609).

### Sample selection.

The Center for Sexual Health (CSH) of the Amsterdam Public Health Service, the Netherlands, is a low-threshold clinic. Clients do not require a referral by a medical doctor, and the consultations are anonymous and free of charge. From November 2018 through December 2019, we invited MSM aged 18 years and older to participate if they: (i) had signs and symptoms suggestive of primary syphilis or secondary stage syphilis; or (ii) were diagnosed with early latent or late latent syphilis (23). In total, 293 men were included in the study. After informed consent, we collected an anal swab, urine sample, peripheral blood sample, and pharyngeal swab, in addition to routine diagnostic samples, such as swabs from pharyngeal-, anal-, or genital ulcer lesions. In this typing study, we included all 287 TP DNA positive samples from 162 participants with at least one TPA DNA positive sample.

For the current analysis TP DNA positive samples were grouped by anatomical locations. Urine samples and genital ulcers were grouped together as “Urogenital.” In a similar fashion pharyngeal swabs and pharyngeal ulcers were grouped as “Pharynx” and anal swabs and anal ulcers were grouped as “Anus.”

### DNA extraction and TP detection.

DNA extraction was performed using multiple optimized methods for each sample type (4). DNA from anal swab, pharyngeal swab, and ulcer swab samples was extracted using isopropanol precipitation (24) or MagNA Pure 24 (Roche Molecular Systems) according to the specifications of the manufacturers. DNA from urine and peripheral blood samples was extracted within 24 h of collection. Into each of two Eppendorf tubes, 1 mL of urine was transferred. After centrifugation at 14,000 rpm for 10 min the supernatant was discarded and the pellet resuspended in 200 μL PBS. DNA from resuspended urine pellets was extracted using isopropanol precipitation (24). DNA extraction from 200 μL of peripheral blood was done using the QIAamp Blood minikit following the protocol from the manufacturer (Qiagen, Hilden, Germany).

The in-house validated real-time qPCR targeting the *polA* gene was performed to detect TP DNA as previously described concerning primers, probes, and amplification protocol (25). A RotorGene Q thermocycler was used (Qiagen, Hilden, Germany) with a standardized fluorescence threshold of 0.04 for determination of Ct values. The PCR was considered positive with a Ct value <36. For samples with Ct value between 36 and 40, DNA was re-extracted and tested again together with the first DNA extract. When the Ct value was ≤40, the result was positive. Ct values >40 were considered negative. In addition, phocine herpesvirus (PhHV) was used as internal control to exclude PCR inhibition. If a sample was inhibited in the PCR, the sample was re-extracted and tested again simultaneously with a 1:10 dilution of the first DNA extract. Due to frequent PCR inhibition of anal swabs, these samples were tested concomitantly at the 1:10 dilution.

### Definition of the clinical syphilis stages.

Primary syphilis was defined as the presence of an ulcer with a positive dark-field microscopy test and/or TP DNA detected with the *polA* PCR. A diagnosis of secondary syphilis was based on typical clinical manifestations such as a rash with or without lymphadenopathy, or mucosal lesions such as condylomata lata, and a RPR titer ≥1:4. Ulcers may also occur in secondary-stage patients. Early latent syphilis was defined as a seroconversion of the CLIA, or a RPR ≥ 1:32, or a 4-fold or higher RPR titer rise in an asymptomatic participant.

### Molecular typing of TP.

The MLST method for the molecular characterization of TP was used as described (6). This method is based on the partial amplification and sequence analysis of genetic regions: *tp0136, tp0548*, and *tp0705*. The allelic profiles consisting of allelic variants per genetic regions, in respective order, constitute the resulting TP-MLST type. Amplification was performed using nested PCR for each genetic region, after which Sanger sequencing was used. Internal primers for genetic region *tp0136* were used if needed. In addition, an extra primer (5’ATCGTTTGTTATGCCGGTTGC) was developed and used, if necessary, for the completion of the 3′ region of *tp0705*. The sequence analysis was done using Bionumerics version 7.6.3 (Applied Maths, bioMérieux). Samples with only one or two out of the three regions typed are referred to as partially typed and were excluded from further analyses.

The lineage of the TP strains (SS14 or Nichols) was determined by the analysis of the sequenced genetic regions of *tp0136* and *tp0548* found in the study. Every new allelic variant was aligned to both references, SS14 and Nichols, using MEGA X version 10.1.8 in order to determine its genetic lineage.

All samples that obtained a TP-MLST type were uploaded in the BIGSdb PubMLST TP database (8) (ID numbers 967 to 1,057) and new allelic variants and new allelic profiles were added to the TP typing scheme with subsequent numbering.

### Data analysis.

Cycle threshold value differences were tested using the independent sample *t* test. Other variables of interest were compared using Fisher’s exact test and Pearson’s chi-square test in SPSS 26.0.0.1 or R 3.6.3 (26). Figures were produced using the packages ggplot2 (27) and VennDiagram (28). To visualize genetic lineages, a phylogenetic tree of the concatenated sequences was generated by first aligning the concatenated sequences using Crustal-Wallis in MEGA X version 10.1.8 followed by the evolutionary analysis using the bootstrapping maximum-likelihood algorithm and the Tamura-Nei method (29) in MEGA X version 10.1.8 (30).
