# Supplementary material for: Treponema pallidum Subspecies Pallidum Intrapatient Homogeneity at Various Body Locations in Men with Infectious Syphilis
Source: Microbiol Spectr. 2022 Jun 23;10(4):e02482-21. doi: 10.1128/spectrum.02482-21 (PMC9430645; doi:10.1128/spectrum.02482-21)
Supplement: Supplemental file 1 — Fig. S1; Tables S1 to S3. Download spectrum.02482-21-s0001.pdf, PDF file, 0.5 MB [file spectrum.02482-21-s0001.pdf]

Supplementary Figure 1

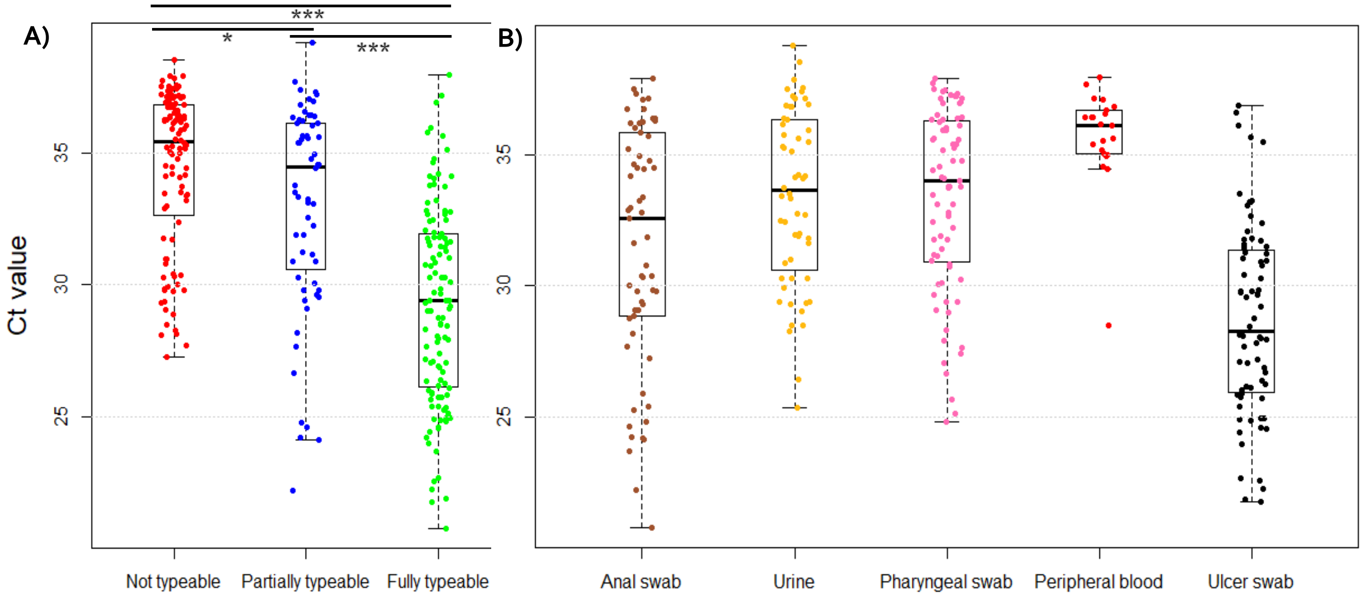

Supplementary Figure 1. Distribution of cycle threshold (Ct) values of all TP DNA positive samples in this study. A) Ct values grouped and colored by typeability. B) Ct values by sample type, of which 61 anal swabs, 56 urine samples, 75 pharyngeal swabs, 22 peripheral blood samples and 73 ulcer swabs. * $p < 0.05$, *** $p<0.0001$ (using one-way ANOVA)

Supplementary Table 1

Supplementary Table 1. Overview of TP DNA positive samples deriving from patients with infectious syphilis and their proportions within each syphilis stage, overall and the number of samples of which a TP type was obtained.

**Type of sample**	**Primary syphilis** 69 patients (%)	**Secondary syphilis** 64 patients (%)	**Early latent syphilis** 29 patients (%)	**Total #typed / Total #samples (%)**
Anal swab	13 (19)	37 (58)	11 (38)	**10 / 61** (16)
Urine	24 (35)	26 (41)	6 (21)	**23 / 56** (41)
Peripheral blood	2 (3)	15 (23)	5 (17)	**1 / 22** (5)
Pharyngeal swab	7 (10)	47 (73)	21 (72)	**35 / 75** (47)
Ulcer swab	63* (91)	10 (16)	0 (0)	**50 / 73** (68)
**Total**	109	135	43	**119 / 287** (42)

**Remaining 6 patients with primary syphilis did not have an ulcer swab available for the study.*

Supplementary Table 2

Supplementary Table 2. Overview of all (partial) allelic profiles of samples per body locations of 182 patients with at least one allelic variant. X refers to an undetermined allelic variant. "Unknown" lineages lack typing information of the *tp0136* and the *tp0548* locus.

Allelic profiles	Lineage	Anus	Urogenital	Peripheral blood	Pharynx	Total
1.3.1	SS14	8	25		9	42
1.1.1	SS14	1	9		9	19
9.7.3	Nichols	2	5	1	8	16
1.1.8	SS14	2	3		3	8
3.2.3	Nichols		4		3	7
1.1.9	SS14		2		1	3
1.66.1	SS14	1			1	2
1.64.1	SS14		1			1
1.52.1	SS14		1			1
1.17.9	SS14		1			1
1.43.1	SS14				1	1
1.65.1	SS14				1	1
30.3.1	SS14				1	1
29.7.3	Nichols		1			1
1.X.1	SS14	3	3		3	9
X.3.1	SS14	2	1		2	5
X.1.1	SS14	1	1	1		3
1.3.X	SS14		1	1		2
1.X.8	SS14			1	1	2
X.1.8	SS14	1				1
X.7.3	Nichols	1				1
29.X.3	Nichols			1		1
1.X.9	SS14				1	1
9.X.3	Nichols		1			1
X.2.3	Nichols		1			1
X.63.1	SS14		1			1
6.X.1	SS14				1	1
X.X.1	Unknown	1		3	4	8
X.1.X	SS14	1	2	2	2	7
1.X.X	SS14	3	2		1	6
X.X.9	Unknown			1	1	2
9.X.X	Nichols			1		1
19.X.X	SS14				1	1
29.X.X	Nichols				1	1
X.66.X	SS14		1			1
X.7.X	Nichols				1	1
X.X.8	Unknown				1	1
X.X.3	Unknown	1				1

Supplementary Table 3

Supplementary Table 3. Chi-squared or Fisher's Exact Tests of distribution of lineages (Nichols and SS14) by (A) syphilis stage, (B) age, (C) HIV status and (D) number of TP DNA detected at different sites of the 93 patients with at least one TP type.

A)		Lineage (N = 93 patients)		
		Nichols n (%)	SS14 n (%)	**Total**
**Syphilis stage**	Primary	7 (15)	41 (85)	48
	Secondary	11 (31)	24 (69)	35
	Early latent	1 (10)	9 (90)	10
	**Total**	19 (20)	74 (80)	93

Fisher's Exact Test 0.13

B)		Nichols n (%)	SS14 n (%)	**Total**
**Age** (in years)	≤34	10 (26)	28 (74)	38
	35-44	4 (15)	23 (85)	27
	45-54	3 (20)	12 (80)	15
	55+	2 (15)	11 (85)	13
	**Total**	19 (20)	74 (80)	93

Fisher's Exact Test 0.72

C)		Nichols n (%)	SS14 n (%)	**Total**
**HIV status**	Negative	4 (15)	23 (85)	27
	Positive	15 (23)	51 (77)	66
	**Total**	19 (20)	74 (80)	93

Pearson's χ^2 test 0.56

D)	#anatomical locations	Nichols n (%)	SS14 n (%)	**Total**
**TP DNA detected**	1	9 (47)	48 (64)	57
	2	6 (31)	10 (14)	16
	3	2 (11)	10 (14)	12
	4	2 (11)	6 (8)	8
	**Total**	19	74	93

Fisher's Exact Test 0.26
